# Loss of function of the *Drosophila* Ninein-related centrosomal protein Bsg25D causes mitotic defects and impairs embryonic development

**DOI:** 10.1242/bio.019638

**Published:** 2016-07-15

**Authors:** Michelle Kowanda, Julie Bergalet, Michal Wieczorek, Gary Brouhard, Éric Lécuyer, Paul Lasko

**Affiliations:** 1 Department of Biology, McGill University, Montréal, Québec H3G 0B1, Canada; 2 RNA Biology Unit, Institut de recherches cliniques de Montréal (IRCM), Montréal, Québec H2W 1R7, Canada; 3 Département de Biochimie, Université de Montréal, Montréal, Québec H3T 1J4, Canada; 4 Division of Experimental Medicine, McGill University, Montréal, Québec H3A 1A3, Canada

**Keywords:** RNA localization, Centrosome, Dynein, Pole plasm

## Abstract

The centrosome-associated proteins Ninein (Nin) and Ninein-like protein (Nlp) play significant roles in microtubule stability, nucleation and anchoring at the centrosome in mammalian cells. Here, we investigate *Blastoderm specific gene 25D* (*Bsg25D*), which encodes the only *Drosophila* protein that is closely related to Nin and Nlp. In early embryos, we find that *Bsg25D* mRNA and Bsg25D protein are closely associated with centrosomes and astral microtubules. We show that sequences within the coding region and 3′UTR of *Bsg25D* mRNAs are important for proper localization of this transcript in oogenesis and embryogenesis. Ectopic expression of eGFP-Bsg25D from an unlocalized mRNA disrupts microtubule polarity in mid-oogenesis and compromises the distribution of the axis polarity determinant Gurken. Using total internal reflection fluorescence microscopy, we show that an N-terminal fragment of Bsg25D can bind microtubules *in vitro* and can move along them, predominantly toward minus-ends. While flies homozygous for a *Bsg25D* null mutation are viable and fertile, 70% of embryos lacking maternal and zygotic Bsg25D do not hatch and exhibit chromosome segregation defects, as well as detachment of centrosomes from mitotic spindles. We conclude that Bsg25D is a centrosomal protein that, while dispensable for viability, nevertheless helps ensure the integrity of mitotic divisions in *Drosophila*.

## Abstract (precis)

**Summary:** In humans, mutations in Ninein or Ninein-like protein result in microcephaly and other severe diseases. We show that while flies lacking the Ninein orthologue can survive, many die as embryos with defects in mitosis.

## INTRODUCTION

Establishment of embryonic patterning in *Drosophila melanogaster* requires localized translation of numerous maternally deposited mRNAs in specific regions of the embryo during the initial nuclear divisions in the syncytial stage of embryogenesis (Lasko, 2012). *Drosophila* primordial germ cells, often called pole cells, are specified by localized posterior determinants, many of which are translated from mRNAs that accumulate in the posterior pole plasm of the oocyte and early embryo. At least 11 mRNAs known to be involved in pole cell development and/or embryonic patterning transiently accumulate in a perinuclear pattern around the pole cell nuclei during nuclear division 9 in embryogenesis, namely *germ cell-less* (Jongens et al., 1992), *polar granule component* (Hanyu-Nakamura et al., 2008), *nanos* (*nos*) (Wang and Lehmann, 1991), *spire* (Dahlgaard et al., 2007), *Tao* (Sato et al., 2007), *arrest* (Parisi et al., 2001), *exuperantia* (Winslow et al., 1988), *oo18 RNA-Binding Protein* (Lantz et al., 1994), *tramtrack* (Read et al., 1992), *cyclin B* (Kadyrova et al., 2007), and *pumilio* (Asaoka-Taguchi et al., 1999; Lécuyer et al., 2007). One of these mRNAs, *nos*, is first anchored to the posterior actin cytoskeleton, and then transported to the migrating posterior nuclei by the motor protein Dynein along astral microtubules (Lerit and Gavis, 2011). Vasa protein (Vas), a DEAD-box helicase essential for germ cell specification, localizes in the same pattern (Lerit and Gavis, 2011). It is assumed that other mRNAs with the same distribution pattern localize through a similar mechanism, although this has not been directly investigated.

In the early *Drosophila* embryo the first ten rounds of nuclear divisions are synchronous and are not coupled to cytokinesis (Foe and Alberts, 1983). During this period, nuclei migrate toward the periphery of the embryo. The next three rounds of nuclear division remain synchronous and uncoupled to cell divisions, except at the posterior pole of the embryo, where nuclei migrate through the germ plasm, slow their divisions and become incorporated within pole cells, the first distinctive cells to form in the embryo. Centrosomes that associate with the nuclei that migrate to the posterior trigger the release of germ plasm components, such as *nos* and Vas, from the embryonic posterior cortex, enabling Dynein-dependent transport into the pole cells as they form (Lerit and Gavis, 2011). This suggests that germ cell specification might be particularly sensitive to the activities of centrosome-associated proteins. Consistent with this, a role for Neurl4, a centrosome-associated protein, in germ cell specification and integrity has recently been revealed (Jones and Macdonald, 2015).

A centrosome typically consists of a pair of centrioles surrounded by pericentriolar material, and it is from this structure that spindle and astral microtubules emanate (Azimzadeh and Bornens, 2007). Centrioles contain nine triplets of microtubules with proximal and distal ends (Vorobjev and Nadezhdina, 1987). The pericentriolar material is a highly organized structure (Fu and Glover, 2012; Lawo et al., 2012). Stringent control of the centrosome and centrioles is vital since abnormalities in spindle pole function can lead to genomic instability. Centrosomes in syncytial *Drosophila* embryos differ in composition from those of other animals; they are considered immature because they are shorter and have no clear difference between their proximal and distal ends (Gonzalez et al., 1998). In addition, centriole duplication occurs after centrosome separation (Callaini and Riparbelli, 1990). This is unlike in mammalian cells where duplication takes place prior to centrosome division (Callaini et al., 1997). Despite these differences, many proteins involved in centrosomal structure and function are conserved between mammals and flies (Woodruff et al., 2014).

In the context of the link between centrosomes and targeting of pole plasm components to the presumptive pole cells, we decided to investigate *Blastoderm specific gene 25D* (*Bsg25D*), because it produces an mRNA that localizes to the posterior pole in a similar pattern to *nos* and the other RNAs mentioned above, and because it encodes a protein related to mammalian Ninein (Nin) and Ninein-like Protein (Nlp), centrosomal proteins involved in microtubule organization (Casenghi et al., 2003; Stillwell et al., 2004). *Bsg25D* was among the first genes to be molecularly characterized in *Drosophila*, and was initially reported to be transcribed only during the blastoderm stage of embryogenesis (Boyer et al., 1987; Roark et al., 1985). However, recent results with more sensitive techniques have shown that, while *Bsg25D* is most abundantly expressed in early embryos, it is also expressed during many developmental stages (Roy et al., 2010; Wasbrough et al., 2010; BDGP *in situ* homepage: http://insitu.fruitfly.org/cgi-bin/ex/insitu.pl)*. Bsg25D* mRNA is both maternally contributed during oogenesis and zygotically expressed in syncytial blastoderm stage embryos (Lécuyer et al., 2007; Tomancak et al., 2007). This transcript is localized to the posterior pole plasm in early embryogenesis and exhibits a prominent perinuclear pattern around the pole cell nuclei during nuclear division 9, similar to *nos* and the other mRNAs discussed above, as well as peri-centrosomal localization in the somatic region of the embryo (Iampietro et al., 2014).

To explore the cellular and developmental roles of *Bsg25D*, we produced loss-of-function mutants and used them to investigate its functions during oogenesis and early embryogenesis. We show that Bsg25D protein and mRNA co-localize to centrosomes and microtubules *in vivo* and that a purified form of Bsg25D protein can bind to microtubules *in vitro*. Furthermore, the localization of *Bsg25D* in oogenesis and embryogenesis is dictated by separable localization elements within the coding region and 3′ UTR, while mislocalization of *Bsg25D* in oocytes affects microtubule polarity and subsequent embryonic patterning. Finally, we find that maternal and zygotic expression of *Bsg25D* is important for full embryonic viability and that mutant embryos frequently exhibit mitotic divisions prior to the midblastula transition (MBT) in embryogenesis.

## RESULTS

### Localization of *Bsg25D* mRNA and protein is dynamic in oogenesis

**Fig. 1.**
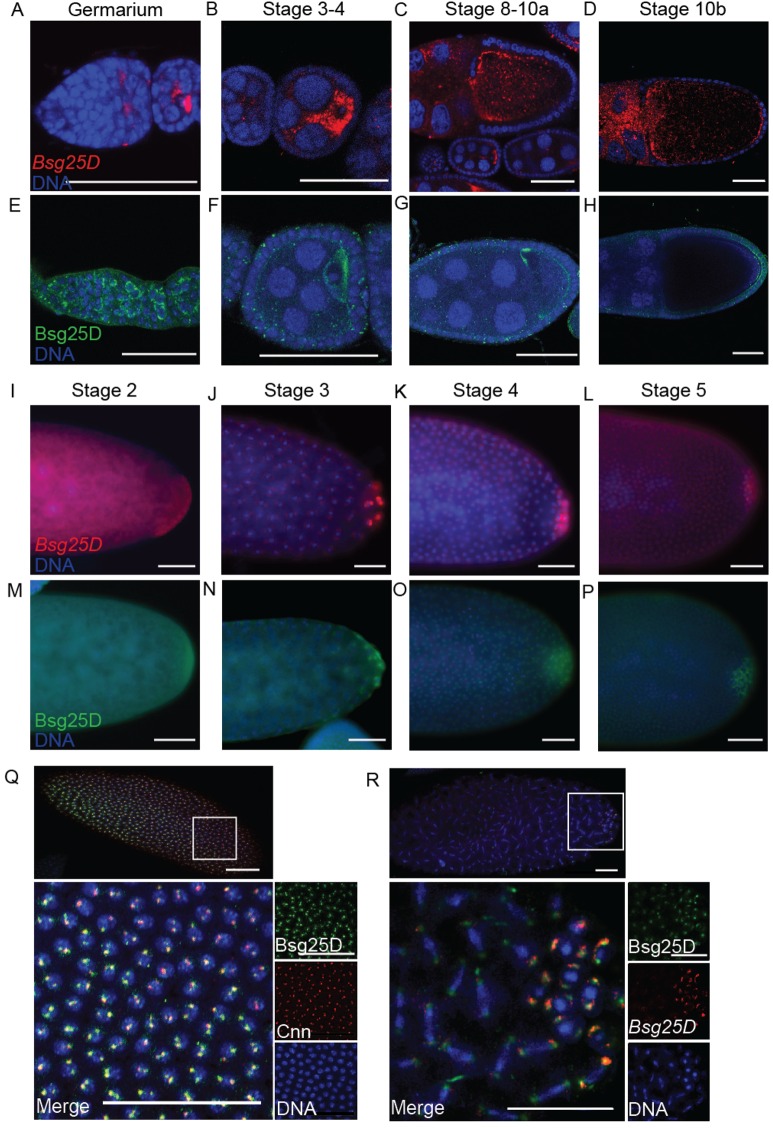
**Localization of *Bsg25D* RNA and protein in oogenesis and early embryogenesis.** (A-D) *Bsg25D* RNA (red) and (E-H) Bsg25D protein (green) share a similar localization pattern in oogenesis. (I-L) Localization of *Bsg25D* RNA (red) and (M-P) Bsg25D protein (green) in stage 2-5 of embryos. Photographs show only the posterior halves of embryos to emphasize the accumulation of *Bsg25D* mRNA and protein in the posterior pole plasm. (Q) High-magnification image showing Bsg25D (green) and Cnn (red) accumulation near the somatic nuclei (blue) of a syncytial blastoderm stage embryo. (R) High-magnification image showing the distribution of *Bsg25D* RNA (red) and Bsg25D protein (green) in posterior the pole cells and mitotic somatic nuclei of a syncytial blastoderm stage embryo. For all images scale bar=50 µm.

We first sought to characterize the distribution of *Bsg25D* mRNA and protein in oogenesis and early embryogenesis as a means of identifying possible sites where its function is required. *In situ* hybridization experiments indicated that *Bsg25D* mRNA is expressed throughout oogenesis. Like many other mRNAs that are ultimately destined for the posterior pole plasm, *Bsg25D* accumulates in the oocyte of stage 2-7 egg chambers. Unlike these others, however, *Bsg25D* mRNA becomes most concentrated at both the anterior and posterior poles of the oocyte from stage 10 onward (Fig. 1A-D). Immunohistochemical experiments using an antiserum that recognizes Bsg25D (Iampietro et al., 2014) revealed that its protein expression largely mirrored that of its mRNA until stage 10, when anterior accumulation of the protein is not as apparent as for the mRNA (Fig. 1E-H).

*In situ* hybridization experiments confirmed previous evidence indicating that *Bsg25D* mRNA is enriched at the posterior of the embryo in its earliest stages of development, and then accumulates in the pole buds and pole cells as they form (Fig. 1I-L). Localization of *Bsg25D* mRNA to the posterior pole plasm is not absolute and some is apparent in somatic regions of the embryo as well, where it accumulates in two puncta on opposite sides of each nucleus (Iampietro et al., 2014; Lécuyer et al., 2007) (Fig. 1J). Immunohistochemical staining showed that Bsg25D protein is distributed in a similar pattern (Fig. 1M-P). To determine the relationship between Bsg25D puncta and centrosomes, we carried out double labeling experiments with antisera recognizing Bsg25D and the expression of centrosomal component Centrosomin fused to GFP (Cnn-GFP). We observed that Bsg25D and Cnn-GFP foci were closely associated, and that the foci of Bsg25D staining are generally larger than those of Cnn-GFP (Fig. 1Q). In many cases the Cnn signal was completely enveloped by the Bsg25D signal. Using a similar approach we also observed colocalization between Bsg25D and γ-tubulin throughout mitosis, but not with α-tubulin (Fig. S1). These results indicate that Bsg25D is a component of *Drosophila* centrosomes, or associates closely with them. Remarkably, *Bsg25D* mRNA also co-localized with centrosomes, both in posterior pole cells and in the somatic region of the embryo (Fig. 1Q,R). The accumulation of both *Bsg25D* mRNA and Bsg25D protein at centrosomes suggests that *Bsg25D* mRNA is translated locally there.

### Generation of *Bsg25D* mutant alleles

**Fig. 2.**
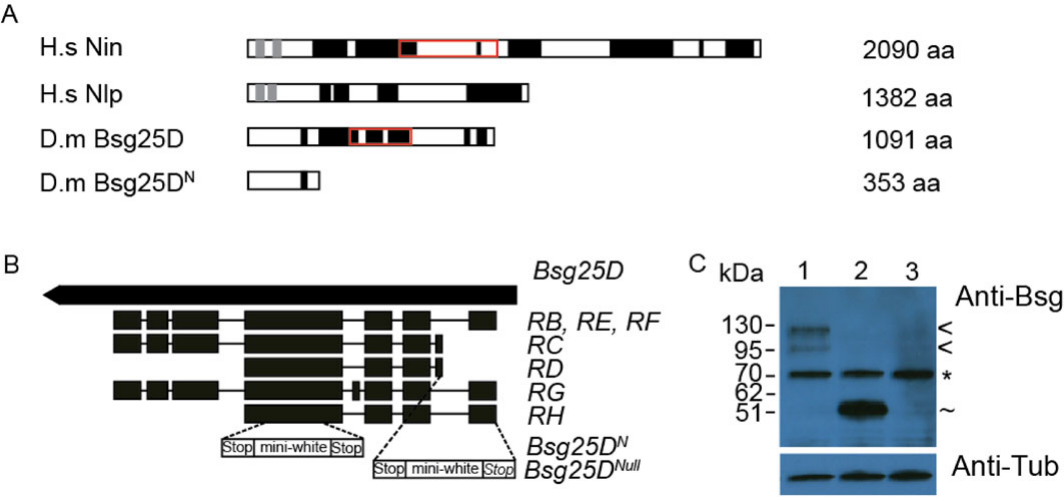
**Mutant alleles of *Bsg25D* generated through gene targeting.** (A) Schematic of Nin, Nlp, Bsg25D and Bsg^N^ proteins. Nin isoform 1 (UniProt identifier Q8N4C6-1) and Nlp isoform 1 (UniProt identifier Q9Y2I6-1), both considered the canonical isoform, and Bsg25D-PB are shown here. EF-hand domains (light grey), coiled-coil domains (black), and the Smc domain (red box) are shown (Dinkel et al., 2016; Hong et al., 2000; Letunic et al., 2006; Sigrist et al., 2012; Wang and Zhan, 2007). (B) Schematic diagram of *Bsg25D*, showing the ORFs of seven different predicted *Bsg25D* transcripts and the sites at which the gene targeting vector pw25.5 was inserted. (C) Immunoblot of ovary lysates from Oregon-R wild-type controls (lane 1), *Bsg25D ^N^*/*Bsg25D ^N^* (lane 2) and *Bsg25D ^Null^/Bsg25D ^Null^* (lane 3). The bands observed correspond to Bsg25D full-length isoforms from different alternatively spliced transcripts (<), a truncated isoform corresponding to the size predicted for *Bsg25D^N^/Bsg25D^N^* (∼) and a non-specific band present in all lysates (*). α-tubulin was used for a loading control.

To investigate *Bsg25D* function, we next used the ends-out gene targeting method (Maggert et al., 2008) to produce premature-termination (*Bsg25D ^N^*, first 353 amino acids) and null (*Bsg25D ^Null^*) alleles of *Bsg25D* (Fig. 2B). Bsg25D, like Nin and Nlp, has numerous coiled-coil domains (Fig. 2A). It also contains a predicted Smc chromosome segregation ATPase domain (Marchler-Bauer et al., 2015) that is also found in Nin, but not in Nlp (Fig. 2A). Nin and Nlp also contain predicted EF-hand domain pairs in their N-terminal regions (Fig. 2A). While substantial sequence similarity between Bsg25D and these proteins is present in their N-termini, some of the conserved residues of EF-hands are absent in Bsg25D. Based on these relationships we consider Bsg25D to be a closer orthologue to Nin than to Nlp. *Bsg25D ^N^* lacks the Smc domain and all but one of the coiled domains (Fig. 2A-C). However, a similar N-terminal fragment of mouse Nin was found to co-purify, in co-immunoprecipitation and pull-down experiments, with γ-tubulin containing complexes (Delgehyr et al., 2005). The *Bsg25D ^Null^* allele does not detectably express Bsg25D protein at all (Fig. 2B,C).

### *Bsg25D* mRNA localization involves elements within both its coding region and its 3′ UTR

**Fig. 3.**
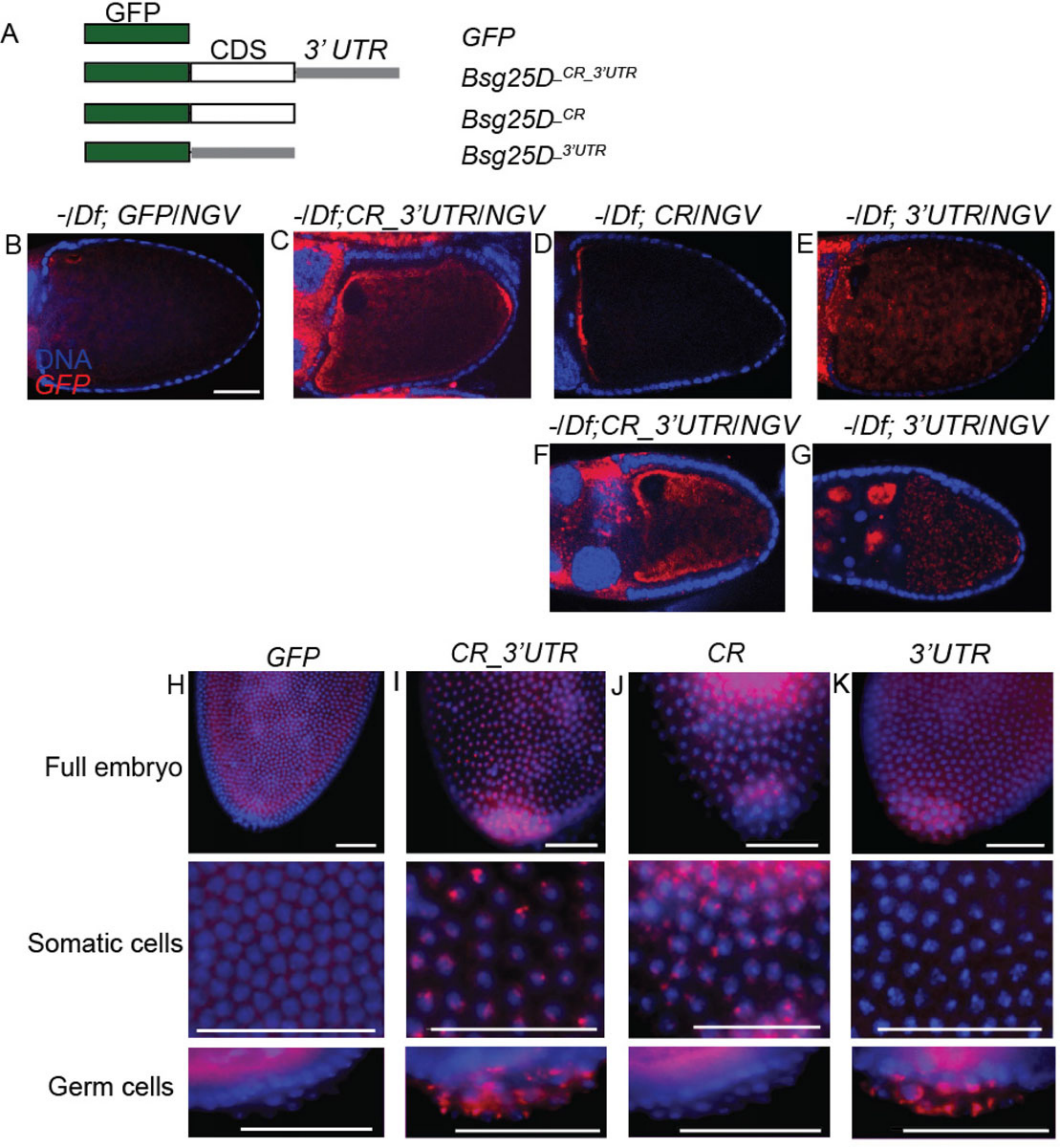
***Bsg25D* RNA contains localization elements within the coding region and 3′ UTR.** (A) Schematic diagram of the transgenic constructs used, eGFP (green), Bsg25D-PB coding region (white), *Bsg25D* 3′ UTR (grey). (B-E) Distribution of transgenically-expressed mRNAs in stage 10 oocytes as shown by *in situ* hybridization, using a probe recognizing *GFP*, in the *Bsg25D ^Null^/Df(2L)6011* (−/Df) genetic background. The coding region alone promotes localization to the anterior pole, while the 3′ UTR promotes posterior localization. (F,G) Posterior localization is not apparent in early stage 10 oocytes expressing full-length *GFP-Bsg25D* (F) but is evident in similar stage oocytes expressing only *GFP* fused to the *Bsg25D* 3′ UTR (G). (H-K) Distribution of GFP-reporter mRNAs in early embryos expressing the transgenes as shown by *in situ* hybridization, using a probe recognizing *GFP*, in a wild-type genetic background. The 3′ UTR is essential for accumulation of these RNAs into the pole plasm and pole cells. All images scale bar=50 µm.

We next aimed to identify potential localization elements in *Bsg25D* mRNA and to investigate whether localization of the mRNA is relevant to its *in vivo* function, we generated a series of transgenic fly lines (Fig. 3A). The genotypes of these flies were confirmed through PCR, northern blot, and immunostaining (Fig. S2A-D). *UASp-eGFP-Bsg25D^ _CR_3′UTR^* (*CR_3′UTR*) flies expressed the full-length coding region of *Bsg25D* (isoform RB) along with the 3′ UTR, *UASp-eGFP-Bsg25D ^_CR^* (*CR*) expressed the full-length coding region but lacked the 3′ UTR, and *UASp-eGFP-Bsg25D ^_3′UTR^* (*3′UTR*) expressed only the 3′ UTR. *UASp-eGFP* (*GFP*) flies, expressing *eGFP* alone, served as a negative control.

Using these transgenic lines, we conducted *in situ* hybridization experiments with an *eGFP* probe to examine the distribution of transgenic *eGFP-Bsg25D* mRNAs in oocytes lacking endogenous *Bsg25D*. As expected, *eGFP* was uniformly distributed in the oocytes of *Bsg25D ^Null^*/*Df*; *GFP*/*NGV* (expressed with *nanos-GAL4-VP16*) females (Fig. 3B)*.* When the full-length *eGFP-Bsg25D* transgene containing the 3′ UTR was expressed (*Bsg25D ^Null^/Df; CR_3′UTR*/*NGV*), *eGFP* distribution faithfully reproduced the pattern of endogenous *Bsg25D* (Fig. 3C, compare with Fig. 1D). In contrast, *eGFP*-*Bsg25D* mRNA containing the coding region alone (*Bsg25D ^Null^*/*Df*; *CR*/*NGV*) was concentrated at the anterior of the oocyte and did not accumulate at the oocyte posterior at stage 10a (Fig. 3D). Conversely, transgenic flies expressing only the *eGFP*-*Bsg25D* 3′ UTR (*Bsg25D ^Null^*/Df; *3′UTR*/*NGV*) precociously localized *eGFP*-*Bsg25D* RNA to the posterior at early stage 10 (Fig. 3E-G). Similar localization patterns are observed when these transgenes are expressed in a wild-type background (Fig. S2E-H).

Next, we examined the distribution of these transgenic mRNAs in otherwise wild-type embryos. In embryos from *NGV*; *GFP* mothers*, eGFP* is found generally in the cytoplasm (Fig. 3H). However, in embryos from flies expressing the *Bsg25D* coding region and 3′ UTR (*NGV*; *CR_3′UTR*), the chimeric mRNA localized to centrosomes in both the germline and the somatic region of the embryo (Fig. 3I). By contrast, *eGFP- Bsg25D ^_CR^* (*NGV*; *CR*) mRNA specifically associated with somatic centrosomes and was largely absent from pole cells (Fig. 3J), while *eGFP- Bsg25D ^_3′UTR^* (*NGV*; *3′UTR*) mRNA was mostly enriched in pole cells (Fig. 3K). This suggests the presence of separable localization elements, one residing in the *Bsg25D* coding region mediating anterior/somatic targeting, and the other within the 3′ UTR of *Bsg25D* directing posterior localization, a pattern established during oogenesis.

### Mislocalization of *Bsg25D* affects microtubule polarity and Gurken deployment in the developing oocyte

**Fig. 4.**
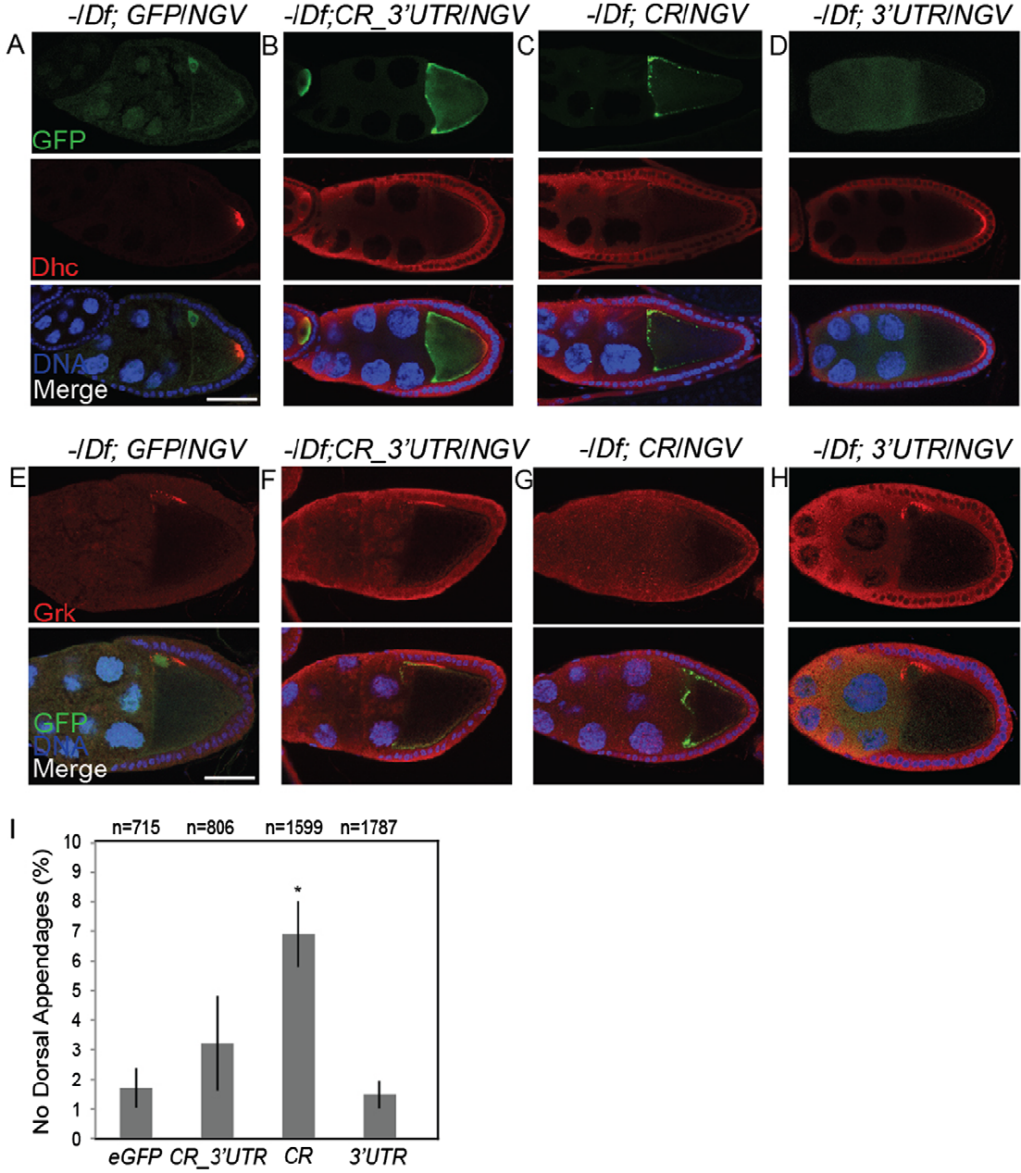
**Ectopic expression of GFP-Bsg25D affects microtubule polarity and Grk deployment.** (A-D) GFP (A) or GFP-Bsg25D (B,C) distribution in the transgenic lines also examined in Fig. 5, in a *Bsg25D ^Null^/Df(2L)6011* (−/Df) genetic background. Note that the anterior accumulation of GFP-Bsg25D protein is more pronounced when the 3′ UTR is absent from the mRNA (compare C with B). (D) GFP protein alone lacking Bsg25D sequences does not stably accumulate at the posterior of the oocyte (top panel). Posterior localization of endogenous Dhc (red) is reduced in oocytes expressing *GFP-Bsg25D* lacking the 3′UTR as compared to other constructs (middle panels, compare C with the others). (E-H) Distribution of Gurken in oocytes expressing transgenic GFP-Bsg25D constructs. Expression of *GFP-Bsg25D* lacking the 3′UTR correlates with reduced accumulation of Grk near the oocyte nucleus (compare G with the others). Scale bar=50 µm. (I) Graph showing the proportion of embryos lacking dorsal appendages produced from females expressing transgenic GFP-Bsg25D constructs. Asterisks indicate statistical significance (*P*<0.05), and graph displays mean±s.e.m.

Ninein family members are involved in microtubule anchoring and nucleation. Therefore, we investigated whether mislocalizing Bsg25D would have an effect on microtubule arrangement in oogenesis, since microtubules are dynamic throughout oogenesis (Parton et al., 2011). We investigated microtubule organization in oocytes expressing the various forms of *eGFP-Bsg25D* used to study localization of its mRNA. First, we compared the distribution of eGFP signal to the distribution of the transgenic chimeric mRNAs. When eGFP protein was expressed on its own (*Bsg25D ^Null^*/*Df; GFP*/*NGV*; Fig. 4A), we detected weak accumulation near the oocyte nucleus, most likely as a consequence of the *K10* terminator element present in the vector. Much more robust targeting of GFP to the oocyte resulted from expression of eGFP-Bsg25D with or without its 3′ UTR, and the distribution of eGFP reflected that of the corresponding mRNA (Fig. 4B,C, compare with Fig. 3C,D). Asymmetric localization of eGFP requires the presence of the Bsg25D coding region, since eGFP alone expressed with the Bsg25D 3′ UTR does not accumulate at the posterior of the oocyte (*Bsg25D ^Null^/Df; 3′UTR/NGV*; Fig. 4D). Immunostaining for Dhc was then used as an indirect means of assessing microtubule polarization; in wild-type oocytes Dhc accumulates at the posterior pole during mid-oogenesis in a microtubule-dependent manner (Palacios and St Johnston, 2002). In a *Bsg25D ^Null^* genetic background, transgenic lines expressing *eGFP*, *eGFP-Bsg25D ^_CR_3′UTR^*, or *eGFP-Bsg25D ^_3′UTR^*, showed correct posterior localization of Dhc (Fig. 4A,B,D). However, when only the coding region of Bsg25D was expressed in fusion with eGFP, (*Bsg25D ^Null^*/*Df*; *CR/NGV*), posterior accumulation of Dhc was reduced (Fig. 4C). The *NGV* driver produced variable levels of expression of *eGFP-Bsg25D^_CR^* in different individual egg chambers, and we observed that expression of *eGFP-Bsg25D ^_CR^* correlated inversely with posterior Dhc localization. With very low expression of *eGFP-Bsg25D^_CR^*, Dhc localization is unaffected (Fig. S3A-C). This suggests that, while loss of *Bsg25D* does not affect microtubule polarity as measured in this assay, ectopic expression and mislocalization of *Bsg25D* may disrupt the polarization of microtubule minus-ends to the posterior pole of the oocyte.

Next, we examined Gurken (Grk) localization in these oocytes, since proper targeting of this protein is dependent on microtubule polarization and on Dynein (MacDougall et al., 2003). In wild-type oogenesis Grk localizes to the antero-dorsal cortex at stage 8, forming a crescent around the oocyte nucleus (Neuman-Silberberg and Schüpbach, 1996). This localization pattern was observed in ovaries from *Bsg25D ^Null^* flies expressing *eGFP*, *eGFP-Bsg25D ^_CR_3′UTR^*, or *eGFP-Bsg25D ^_3′UTR^* (Fig. 4E,F,H,J). Grk localization is reduced in stage 8 egg chambers expressing *eGFP-Bsg25D ^_CR^* (Fig. 4G), although like Dynein, the intensity of localized Grk inversely correlates with the level of *eGFP-Bsg25D ^_CR^* expression (Fig. S3D). There also appeared to be posterior extension of the Grk domain in some oocytes expressing *eGFP-Bsg25D ^_CR^* (Fig. 4G), although variable expression of the transgene, as well as variability in the Grk domain even in wild-type egg chambers (Caceras and Nilson, 2005), makes it difficult to draw firm conclusions about the robustness of this phenotype. We also observed that more embryos produced by females expressing *eGFP-Bsg25D ^_CR^* lacked dorsal appendages, as compared to those produced from wild-type females or from females expressing any of the other transgenic constructs (Fig. 4I). This phenotype could be rescued by endogenous *Bsg25D* (Fig. S3E-F). This suggests that the persistence of Bsg25D at the oocyte anterior may result in a failure to properly target Grk during mid-oogenesis, affecting dorsal appendage formation.

### Bsg25D can bind microtubules, and with Dynein can move along microtubules toward their minus-ends, *in vitro*

**Fig. 5.**
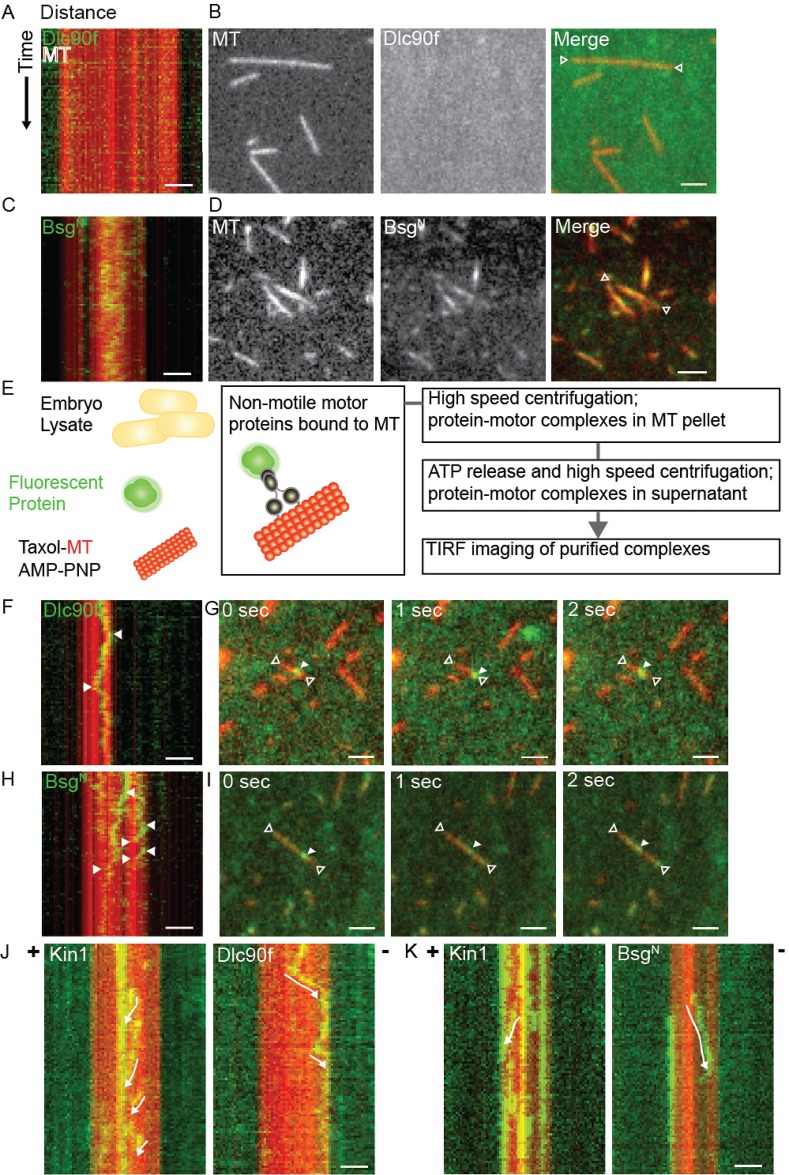
**Bsg25D is a microtubule binding protein.** (A,B) TIRF imaging of purified Alexa Fluor 488-labelled GST-Dlc90f (green) and tetramethylrhodamine-labelled bovine microtubules (red) in BRB80 buffer. No binding is observed either in the kymograph (A) or in direct images (B). (C,D) TIRF imaging of purified Alexa Fluor 488-labelled GST-Bsg^N^ (green) and tetramethylrhodamine-labelled bovine microtubules (red) in BRB80 buffer. Binding is evident both in the kymograph (C) and in direct images (D). (E) Schematic diagram of the microtubule pull-down assay used to purify motor proteins from *Drosophila* embryo lysate, a detailed description is provided in Materials and Methods. (F,G) With addition of purified motor proteins, movement events along microtubules were recorded for GST-Dlc90f in (F) a kymograph (arrows point to the beginning and end of a movement) and in (G) a series of still images from a movie. In (G) the open arrows point to the ends of a microtubule, while the closed arrow points to a GST-Dlc90f molecule. (H,I) With addition of purified motor proteins, movement events along microtubules were also recorded for GST-Bsg^N^ in (H) a kymograph and in (I) a series of still images from a movie. Arrows are as in F,G. (J,K) Kymographs comparing movement events of (J) GST-Dlc90f and (K) His-Bsg^N^ with those of Kinesin-1 imaging to indicate direction of movement. Movements are indicated with white arrows, GST-Dlc90f and GST-Bsg^N^ move in the opposite direction to Kinesin-1. In all images, scale bar=2 µm.

Next, we explored the dynamics of Bsg25D association with microtubules *in vitro*. To determine whether Bsg25D can associate with microtubules, we used total internal reflection fluorescence microscopy (TIRF) to conduct live imaging of purified Bsg25D and microtubules (Gell et al., 2010). For these experiments, because we were unable to express full-length Bsg25D in bacteria despite repeated efforts, we used Bsg^N^, the truncated form of Bsg25D containing only the N-terminal 353 amino acids (Fig. S4). We found that purified Bsg^N^ alone in BRB80 buffer bound efficiently to microtubules, unlike the control protein Dynein light chain 90f (Dlc90f), which requires Dynein intermediate chain (Dic) and Dynein heavy chain (Dhc) to bind to microtubules (Fig. 5A-D; Movies 1, 2) (Song et al., 2007). Bsg^N^ bound diffusely to microtubules, with no specific preference for minus- or plus-ends (Fig. 5C,D), and no binding events were ever observed for Dlc90f (Fig. 5A,B). Next, we used a microtubule pull-down assay (modified from Amrute-Nayak and Bullock, 2012; Lindesmith et al., 2001) to purify motor proteins from *Drosophila* embryo lysates (Fig. 5E). These isolated motor protein complexes were then imaged by TIRF microscopy to observe potential transport of Dlc90f and Bsg^N^ protein molecules. In this manner, we detected transport of both Dlc90f (Fig. 5F,G; Movie 3) and Bsg^N^ (Fig. 5H,I; Movie 4). Movement events were observed more frequently for Bsg^N^ than for the control protein Dlc90f (Fig. 5D). Furthermore, purified motor protein Kinesin-1 (Kin1) was used to indicate microtubule plus-ends after Bsg25D^N^ imaging. This was done by flowing buffer into the channel to wash away Bsg25D^N^ protein-motor protein complexes, and subsequently flowing into the same channel Kin1 and 1 mM ATP. In this way we determined that the movement of these proteins is in the minus-end direction, suggesting this movement is Dynein-dependent for Dlc90f (Fig. 5J; Movies 5, 6), and Bsg^N^ (Fig. 5K; Movies 7, 8).

We measured the speed of Kin1 movement as 0.864±0.047 μm s^−1^ (means±s.e.m.; *n*=34), which is consistent with earlier measurements of its *in vitro* velocity (Howard et al., 1989). We also found that Dlc90f and Bsg^N^ moved at nearly identical speeds of 0.983±0.109 μm s^−1^ (*n*=23) and 0.978±0.074 μm s^−1^ (*n*=30), respectively, and these measurements are consistent with earlier analyses of Dynein movement in 1 mM ATP (Paschal et al., 1987; Ross et al., 2006).

### Bsg25D functions *in vivo* to ensure accurate chromosome segregation during early embryonic nuclear divisions

**Fig. 6.**
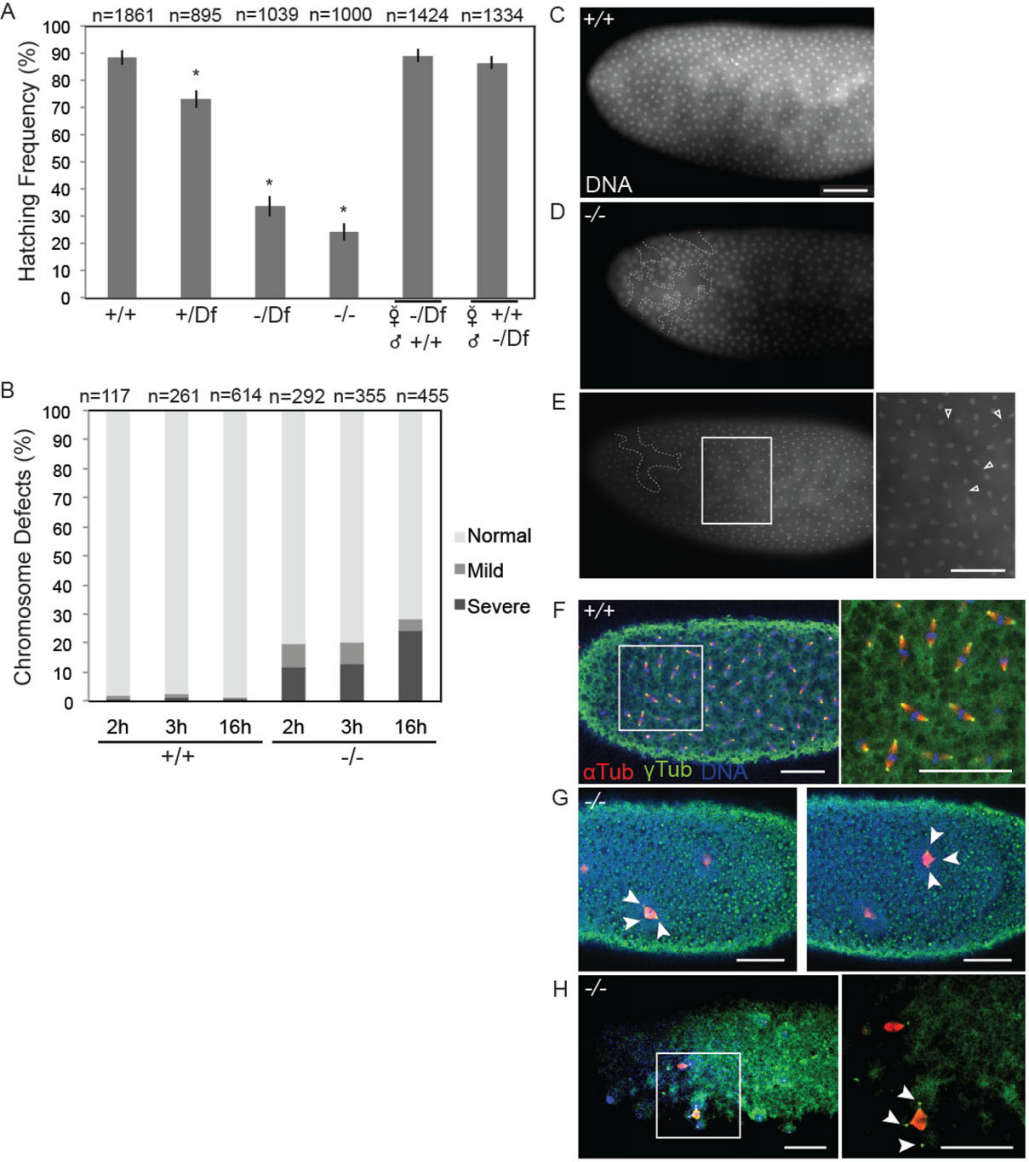
***Bsg25D* mutant embryos have a reduced hatching rate and exhibit mitotic defects.** (A) Graph comparing frequency of hatching for embryos produced by females of the following genotypes crossed to males of the same genotype: Oregon-R (+/+), Df(2*L*)6011 heterozygotes (*+/Df*), *Bsg25D ^Null^/Df(2L)6011 (−/Df*), and *Bsg25D ^Null^/Bsg25D ^Null^* (*−/−*). Asterisks indicate statistical significance (*P*<0.05) and data is displayed as mean±s.e.m. Embryonic viability is recovered when either *Bsg25D ^Null^/Df(2L)Exel6011* females were crossed to wild-type males, or wild-type females were crossed to *Bsg25D ^Null^/Df(2L)Exel6011* males. (B) Graph showing the frequency of Oregon-R (+/+) and *Bsg25D ^Null^* (−/−) embryos exhibiting mitotic errors at three developmental time points. (C) DAPI staining of a wild-type embryo illustrating a typical regular arrangement of syncytial nuclei. (D,E) Examples of embryos graded as having mild mitotic defects. (D) An embryo with a region of nuclear fallout (white dashed line) and (E) an embryo with numerous anaphase bridges (arrows). (F) Anti-α-tubulin, anti-γ-tubulin immunostaining and DAPI staining of a wild-type embryo showing mitotic spindles. (G,H) Examples of embryos graded as having severe mitotic defects. (G) Two images of the same embryo showing two tripolar mitotic divisions (arrows) with γ-tubulin attached to mitotic spindle and (H) an embryo showing a tripolar spindle with γ-tubulin delocalized from mitotic spindle. Inset, with DAPI channel removed for better visualization of α-tubulin and γ-tubulin. In all images scale bar=50 µm.

Our analysis of hemizygous flies, in which the *Bsg25D ^Null^* allele was combined with a deficiency chromosome, *Df(2L)Exel6011*, which deletes the *Bsg25D* locus, revealed that *Bsg25D ^Null^*/*Df(2L)Exel6011* flies are viable and sufficiently fertile to be maintained as a stock. To evaluate more clearly whether *Bsg25D* loss-of-function impacts embryonic development, we next performed quantitative embryo viability assays following different crossing schemes. We first examined embryos from *Bsg25D ^Null^*/*Df(2L)Exel6011* females crossed to *Bsg25D ^Null^*/*Df(2L)Exel6011* males, which lack both maternally- and zygotically-expressed *Bsg25D*, and found however that approximately 70% failed to complete embryogenesis and did not hatch (Fig. 6A). We obtained similar results from embryos produced by *Bsg25D ^Null^*/*Bsg25D ^Null^* females crossed to *Bsg25D ^Null^*/*Bsg25D ^Null^* males, and from *Bsg25D ^N^*/*Bsg25D ^N^* females crossed to *Bsg25D ^N^*/*Bsg25D ^N^* males. Complete viability was recovered when *Bsg25D ^Null^/Df(2L)Exel6011* females were crossed to wild-type males or when wild-type virgin females were crossed to *Bsg25D ^Null^/Df(2L)Exel6011* males (Fig. 6A). We conclude that *Bsg25D* function is required for embryonic development and that either the maternal contribution of *Bsg25D*, or its early zygotic expression, is sufficient for its function in embryogenesis.

We next examined the initial nuclear divisions in *Bsg25D ^Null^/Bsg25D ^Null^* embryos by staining their chromosomes with DAPI. This revealed that many such embryos exhibit excessive nuclear clearance from the embryo cortex (nuclear fallout) compared to wild-type controls (Fig. 6C,D). Moreover, *Bsg25D ^Null^/Bsg25D ^Null^* embryos showed abnormal nuclear aggregates, both large and small, and bridges between chromosomes, indicating failed chromatid separation (Fig. 6B,E). These defects in nuclear division ranged from mild (Fig. 6B-E), where the normal uniform pattern of nuclear divisions continues through the usual 13 rounds despite the phenotypes described above, to severe, where nuclear divisions fail resulting in embryonic lethality (Fig. 6B,G,H). Severely affected embryos do not cellularize and often display monopolar (not shown) and tripolar spindles (Fig. 6G,H). Many embryos were observed to have chromosome segregation defects in their initial nuclear cycles; however, in these early stage embryos the centrosomal marker γ-tubulin appeared to be localized normally, exhibiting a tight association with the mitotic spindle, as seen in wild-type specimens (Fig. 6F,G; Movie 9). In slightly older *Bsg25D ^Null^* embryos, which had undergone more nuclear cycles, delocalization of the centrosome from the mitotic spindle can be observed (Fig. 6H; Movie 10). This suggests that a subset of *Bsg25D ^Null^* embryos fail to properly anchor centrosomes to the mitotic spindle, which may contribute to embryonic lethality.

Finally, to determine whether pole cell specification is particularly sensitive to *Bsg25D* function, we counted pole cells from embryos lacking *Bsg25D* that developed as far as the cellular blastoderm stage and from control embryos. We observed a modest decrease in pole cell number in progeny embryos from crosses among *Bsg25D ^Null^*/*Df(2L)Exel6011* male and female flies that was statistically significant with respect to wild-type controls (29±0.81 vs 34±1.97, *P*=0.010). When we made other similar comparisons, we also observed small decreases consequent to loss of *Bsg25D* activity that were however not statistically significant (*Bsg25D ^Null^*/*Df(2L)Exel6011*; *GFP/NGV*, 29±1.87 pole cells, *P*=0.056 when compared with wild-type, and *Bsg25D ^N^/Bsg25D ^N^*, 30±1.58 pole cells, *P*=0.102 when compared with wild-type). We therefore cannot conclude that *Bsg25D* has a particular function in pole cell specification. However, since a significant number of embryos lacking *Bsg25D* activity are unable to complete more than nine rounds of nuclear divisions, and consequently do not reach the stage of development when pole cells would form, we could not include such embryos in our analyses.

## DISCUSSION

In this work, we observed that a purified fragment of Bsg25D can bind to microtubules *in vitro*, and in the presence of purified motor proteins, move along them primarily in the minus-end direction at a velocity similar to that of Dynein. An association between Bsg25D and Dlc90f was previously identified in a high-throughput protein-protein interaction study (Giot et al., 2003). Our results are also consistent with results obtained with both Nin and Nlp, which bind to cytoplasmic Dynein through their N-termini (Casenghi et al., 2005). Targeting Nin and Nlp to the centrosome occurs through Dynein-mediated transport and is dependent on the microtubule cytoskeleton (Casenghi et al., 2005). While the overall direction of Bsg25D^N^ movement was minus-end directed, we also documented instances of movement toward the plus-end of a microtubule. This is consistent with imaging data from live mammalian epithelial cells demonstrating bidirectional microtubule-directed movement for Nin (Moss et al., 2007). Bidirectional movement has been observed in other single molecule assays and is a well-established characteristic of Dynein (Amrute-Nayak and Bullock, 2012; Reck-Peterson et al., 2006). One potential difference between Bsg25D and its mammalian counterparts is that our *in vitro* experiments indicate that Bsg25D binds to microtubules in the absence of Dynein, which has not been demonstrated for Nin or Nlp. More targeted experiments could in the future establish the mechanistic relationship between Bsg25D and Dynein. These could include depleting for Dynein the motor protein complexes used for the TIRF imaging and determining whether there are effects on Bsg^N^ mobility, or mapping and mutating the site on Bsg^N^ necessary for Dynein binding, and then determining whether such a mutated protein can move along microtubules.

While Bsg25D has extensive sequence similarity to Nin and Nlp, it appears to be more closely related to Nin. Like both Nin and Nlp, Bsg25D contains numerous coiled-coil domains. In addition, one of three cAMP-dependent protein kinase (PKA) phosphorylation sites in Nin is conserved in Bsg25D (amino acids 124-130). This may be important for Bsg25D function, as Nin phosphorylation has been linked for centrosomal localization of certain Nin isoforms, and phosphorylation by PKA has been found to play a critical role in mitotic progression (Chen et al., 2003; Hong et al., 2000; Kotani et al., 1998; Lin et al., 2006). Bsg25D shares a conserved D-box domain with Nlp that is not present in Nin (Bsg25D amino acids 268-276), but it does not have the D-box or Ken-box motifs that for Nlp have been experimentally shown to be important for cell cycle dependent degradation (Nlp amino acids 633-641 and 495-497) (Wang and Zhan, 2007). Nlp has phosphorylation sites for Aurora B or Cdc2/cyclin B1 kinases that do not appear to be conserved in Bsg25D, however independent mass spectrometry analyses of Nlp and Bsg25D reveals the presence of many phosphoserine and phosphothreonine residues at similar locations within both proteins (Casenghi et al., 2003; Zhai et al., 2008). Nlp phosphorylation by Plk is required for Nlp dissociation from the Dynein-Dynactin complex allowing for cell cycle progression in human cell lines (Casenghi et al., 2005).

While *Bsg25D* mutants can survive to become fertile adults, we observed that a majority of embryos that lack maternal and zygotic Bsg25D fail to hatch, and exhibit mitotic defects ranging from mild to very severe. Our experiments do not allow us to distinguish whether the mitotic defects are a cause or a consequence of the failure of many such embryos to develop. However, since Bsg25D associates with centrosomes, we can hypothesize that it contributes to their functions in microtubule nucleation and/or anchoring, and that its loss may cause abnormal mitotic spindle formation. While this manuscript was under review, we became aware of another study of Drosophila *Bsg25D* (Zheng et al., 2016). That paper reports similar results to ours with respect to localization of Bsg25D to centrosomes in early embryos. While both studies found *Bsg25D* null mutants to be viable, in contrast to our results their *nin^1^* allele did not produce a significant decrease in embryonic viability. This quantitative difference in our results could be a consequence of differences in culture conditions or genetic backgrounds.

A role for mammalian Nin in connecting microtubules to the centrosome has been proposed (Shinohara et al., 2013). As well, siRNA-mediated knockdown of Nin in human immortal cell lines resulted in mitotic catastrophe, cell cycle arrest in G2/M phase and apoptosis (Kimura et al., 2013). Given this severe phenotype, it is surprising that *Bsg25D* function is not required for viability in *Drosophila* under laboratory conditions, especially since only one Ninein-related protein is present in flies as opposed to two in mammals. In humans the rare disease Seckel syndrome-7 (SCKL7) is caused by missense mutations in the *NIN* gene (Dauber et al., 2012). SCKL7 results in a growth phenotype called microcephalic primordial dwarfism, which is a severe form of growth failure wherein growth restriction occurs *in utero* and continues after birth (Bober et al., 2010; Dauber et al., 2012). These patients, however, often survive until adulthood. Furthermore, Nlp has been linked to ciliopathies, Usher syndrome and Leber congenital amaurosis (van Wijk et al., 2009). Knockout mice for both *Nin* and *Ninl* (which encodes Nlp) have been prepared (Brown and Moore, 2012) but they have not yet been studied in detail.

In *Drosophila* we also observed defects in Dhc and Grk localization upon overexpression and mis-localization of *eGFP-Bsg25D* mRNA in developing oocytes, which in turn led to an altered distribution of eGFP-Bsg25D protein. Polarization of microtubules within the developing oocyte is critical for transport of mRNAs necessary for axis determination in the early embryo. Our data suggest that overexpression and/or mislocalization of Bsg25D during oogenesis may affect microtubule-dependent localization processes, such as *grk* (MacDougall et al., 2003), and Dhc localization (Li et al., 1994), within the oocyte. Consistent with this, ubiquitous expression of *Bsg25D* with actin or tubulin Gal4 drivers results in early pupal lethality, also demonstrating that overexpression of Bsg25D is deleterious (Zheng et al., 2016).

Analogous results have been obtained for Nin and Nlp in mammalian cells. For example, in mammalian cultured cells overexpression of Nlp recruits γ-tubulin and hGCP4, a component of the γ-tubulin ring complex (γ-TURC) to ectopic loci, resulting in off-site microtubule nucleation and spindle formation (Casenghi et al., 2003). Overexpression of Nin has also been reported to lead to mis-localization of γ-tubulin in cultured human cells (Stillwell et al., 2004). Nlp overexpression is also frequently associated with cancer, including head and neck squamous cell carcinomas and ovarian cancer (Qu et al., 2008; Yu et al., 2009). In one study Nlp was found to be overexpressed in 80% of human breast and lung carcinomas that were investigated, and its overexpression led to tumorigenesis in transgenic mice (Shao et al., 2010).

In conclusion, our study of the dynamics of Bsg25D *in vitro* and of the consequences of its mutation or ectopic expression in an intact metazoan establish *Drosophila* as a model system for studying Ninein family proteins. Further work in this system will help reveal potential mechanisms through which loss or gain of function of Nin and Nlp might result in human disease.

## MATERIALS AND METHODS

### Drosophila strains

Oregon-R was used as wild-type for all experiments. The deficiency spanning *Bsg25D* (*Df(2L)Exel6011*, BL#7497) was received from the Bloomington *Drosophila* Stock Center. Truncated and null alleles of *Bsg25D* were generated using the ends-out gene targeting method, using the pw25.5 vector (generously provided by Dr David R. Hipfner; Maggert et al., 2008). Primers used to produce *Bsg25D ^N^* from *D. melanogaster* genomic DNA were: left arm Forward Not1-tkv-Bsg 5′-GCGGCCGCCATCGACGCGGTATCGATATTC-3′ and Acc651-tkv-Bsg 5′-GGTACCCTAACAGAGGAGAGCCCTCG-3′, and right arm Asc1-Bsg-Bsg 5′-GGCGCGCCCCACGGCAAGCAAAGCCAC-3′ and Reverse Asc1-Bsg-Bsg 5′-GGCGCGCCGCGATAGAAACGTGTTGTTGGG-3′. Primers for *Bsg25D ^Null^* were: left arm Forward Acc651-Bsg-Bsg 5′-GGTACCGGTAGCCACCTAAGATCCATAC-3′ and Reverse Not1-Bsg-Bsg 5′-GCGGCCGCCAATCGGCTATCTCTCCCTC-3′, and right arm Forward Asc1-Bsg-Bub1 5′-GGCGCGCCGGTTACGGATAATGGAGGTATC-3′ and Reverse Asc1-Bsg-Bub1 5′-GGCGCGCCCTTGAGCGCCACTACATTGC-3′. *UASp-eGFP-Bsg25D* transgenic flies were generated as described in Iampietro, et al. (2014). Briefly, the *Bsg25D ^_CR_3′UTR^* (*Coding Region+3′ UTR*), *Bsg25D ^_CR^* (*Coding region*) and *Bsg25D ^_3′UTR^* (*3′ UTR*) sequences were amplified by PCR using *Drosophila* Gene Collection bacterial cDNA clones (dBsg25D=LD21844) as template and primers listed below containing the restriction sites required for the insertion in the vector. PCR fragments were ligated into the pGem4-GFP vector to generate in-frame fusion cassettes with the GFP coding region in 5′ to Bsg25D sequences. The GFP-fusion cassettes were then sub-cloned into the pUASTp-attb plasmid (generously provided by Dr Howard Lipshitz, Molecular Genetics Department, University of Toronto, Ontario, Canada) by using the *Eco*RI restriction enzyme to obtain the transgenesis vectors. All vector sequences were confirmed by sequencing and injected into syncytial stage embryos of the attP-3B acceptor fly line (stock number BL24871) using a Leica DMIL microinjection microscope. Subsequent selection of transgenic progeny was performed as described previously (Bischof et al., 2007; Venken et al., 2006), using primers Bsg25D CR Fw- Kpn1 5′-ATTAGGTACCATGGAGGTATCCGCCGATCCG-3′ and Bsg25D CR Rv EcoR1 5′-ATTAGAATTCCTAAGGCATGCCAGGCAGTCC-3′, and Bsg25D 3′UTR Fw Kpn1: 5′-ATTAGGTACCTAGTTTGCCCCACCGGCAAAC-3′ and Bsg25D 3′UTR Rv EcoR1: 5′-ATTAGAATTCTCGAAAGTATTGATTTAAGCACTGA-3′.

### Immunoblots

Ovaries for immunoblotting were dissected from 2-5-day-old females, lysed in ovary lysis buffer (1× PBS, 1× Halt proteinase inhibitor, 1% PMSF in water) and loaded on an 8-15% gradient gel. The primary antibodies for immunoblotting were: guinea pig anti-Bsg25D (Iampietro et al., 2014; 1:20,000), mouse anti-α-tubulin (Sigma #T6199, 1:10,000), mouse anti-Dynein heavy chain (Developmental Studies Hybridoma Bank #2C11-2, 1:1000), and mouse anti-GFP (Molecular Probes monoclonal antibody 3E6, 1:2500). Secondary antibodies used were donkey anti-guinea pig (Jackson Labs #706-035-148, 1:2500) and anti-mouse (GE Healthcare, #NA931, 1:5000 dilution).

### Immunofluorescence and *in situ* hybridization

Embryos were collected as previously described (Lécuyer et al., 2008). Primary antibodies used for immunostaining were; guinea pig anti-Bsg25D (1:2000), mouse anti-Dynein heavy chain (1:500), rabbit anti-γ-Tubulin (Sigma #T0950, 1:50), rat anti-α-Tubulin (AdB Serotec #MCA78G, 1:50), and rabbit anti-Grk (Dehghani and Lasko; 2015; 1:500). For DNA staining DAPI (Invitrogen #D3571) was used at 10 μg/ml. Secondary antibodies were goat anti-guinea pig (Abcam Dylight 488 #ab96959, 1:500) goat anti-mouse, rabbit and rat (Thermo Fisher Scientific, A11030, A11010 and A-11081, 1:500). Images were collected on the Zeiss LSM510 confocal laser-scanning microscope at the CIAN, Department of Biology, McGill University.

*In situ* hybridization and co-staining was performed as previously described (Lécuyer et al., 2008; Iampietro et al., 2014). Bsg25D anti-sense RNA probe was synthesized from clone LD21844 of the *Drosophila* gene collection library and a full-length probe was used.

Embryos and ovaries for immunostaining or *in situ* were fixed with 4% paraformaldehyde, except for embryos stained for anti-γ-tubulin and anti-α-tubulin. For γ- and α-tubulin stainings embryos were dechorionated with bleach and shaken in heptane for 30 s, followed by shaking for 30 s in 50% heptane:50% methanol to crack the vitelline membranes. Finally, embryos were placed on a nutator for 1 h in methanol, then rehydrated for staining or kept at −20°C for future use.

### Embryo quantification

For hatching counts virgin females were mated to young males on grape juice plates containing yeast. Total eggs from overnight collections were counted and then aged for 48 h at 25°C. After 48 h unhatched eggs were counted. For DNA damage counts embryos were collected for 2, 3 and 16 h, fixed and stained with DAPI (Lécuyer et al., 2008). Embryos were counted manually on a Leica DM6000B microscope under a 20× objective.

### *In vitro* microtubule assays

Bovine tubulin was purified and tetramethylrhodamine labeled as previously described (Noujaim et al., 2014; Wieczorek et al., 2015). Paclitaxel stabilized microtubules used for microtubule pull-down assay were polymerized as described previously (Noujaim et al., 2014). TIRF imaging of microtubules was performed on a Zeiss Axiovert Z1 microscope chassis, using a 100×1.45 NA Plan-apochromat objective lens, and Zeiss TIRF III slider.

GST-tagged full length Dlc90f (39 kDa) and His-tagged N-terminal Bsg25D (43 kDa) protein was expressed and purified from bacteria*.* Both proteins were fluorescently labeled with Alexa Fluor 488 TFP ester (Thermo Fisher Scientific Cat: A37570). A size exclusion column (Amicon Ultra-0.5 30K, UFC503024) was used to concentrate dye-protein conjugates.

A microtubule pull-down assay modified from (Amrute-Nayak and Bullock, 2012; Lindesmith et al., 2001) was used to purify motor proteins from *Drosophila* embryo lysate. *Drosophila* embryos were collected for 4 h, lysed in BXB buffer (Amrute-Nayak and Bullock, 2012), and mixed with purified fluorescent protein along with an AMP analog, AMP-PNP. Isolation of microtubule-motor protein complexes was completed by centrifugation in a Beckman Airfuge at maximum speed, the pellet containing these complexes was washed with assay buffer and the supernatant with unbound protein was discarded. Assay buffer containing ATP resulted in a release of motor protein from microtubules. These isolated motor protein complexes were then mixed with anti-bleach buffer and imaged by TIRF microscopy (Amrute-Nayak and Bullock, 2012). Rat Kinesin-1 430-GFP and purified bovine brain tetramethylrhodamine-labeled α- and β-tubulin were prepared as previously described (Noujaim et al., 2014). MetaMorph was used to record live imaging and analysis. Velocities were calculated using MetaMorph and shown as mean±s.e.m.
